# A New View of Pterosaur Feeding Habits

**DOI:** 10.1371/journal.pbio.0050217

**Published:** 2007-07-24

**Authors:** Liza Gross

Few things capture a child’s imagination like the age of dinosaurs, and it’s no wonder. The dinosaur seems an improbable beast, yet the fossil record tells us that vegetarian sauropods longer than a football field once lumbered alongside tractor-trailer–sized predatory carnosaurs. Above these behemoths soared the reptilian pterosaurs, some propelled by wings spanning more than 12 meters. Although pterosaurs are not dinosaurs, the two groups may well share a common ancestor on the reptilian tree. The size and body plan of these long-extinct animals can be reliably reconstructed from the fossil record, as can the dates of their time on Earth. But fossils only rarely provide direct evidence of behavior and ecology, freeing children to imagine whatever wild scenarios they please and forcing researchers to develop innovative approaches to recreate the ecology of an ancient era.

One way scientists gain insight into ecological traits of extinct animals is by comparing fossilized morphological features to those of living animals. Such studies have suggested that some pterosaurs may have fed like modern-day “skimmers,” a rarified group of shorebirds, belonging to the genera *Rynchops*, that fly along the surface of still bodies of water scooping up small fish and crustaceans with their submerged lower jaw. Structural similarities between pterosaur and *Rynchops* skulls and jaws suggested to some paleontologists that certain pterosaur taxa were anatomically suited for skimming. But the fossilized jaws of putative pterosaur skimmers also bear features that undercut this hypothesis, including pointed jaw tips that likely couldn’t deflect water, and thus reduce energy costs, the way the blunted jaw tips of *Rynchops* presumably do.

In a new study, Stuart Humphries et al. report that biomechanical analyses offer valuable insights into the feasibility of pterosaur skimming. By combining experiments using life-size models of jaws from postulated pterosaur and model skimmers with hydrodynamic and aerodynamic modeling, the researchers show that skimming requires more energy than giant reptilian fliers were likely able to supply.

Though considered reptiles, pterosaurs appear to lack scales and were, in fact, covered in short fur. Their wings, like those of bats, comprised a sophisticated flight membrane supported by elongate wing bones. Their extremely light, hollow skeletons were presumably filled with air, allowing these gigantic animals to take to the skies, which they did as far back as 230 million years ago. Guided by specimens and data on jaw morphology, the researchers built model bills of a *Rynchops* subspecies (*R. niger cinerascens*), a suggested pterosaur skimmer (*Thalassodromeus sethi*), and a presumed nonskimming, smaller pterosaur (*Tupuxuara* sp.). The models were suspended from a trolley and towed along a water-filled trough at speeds documented for *Rynchops* skimmers. The models were rigged with instruments designed to measure strain—the physical response to an applied force like water—which was used to calculate the drag force acting on the models as they “skimmed” the water.

In the skimming experiments, the two pterosaur model bills experienced nearly ten times as much drag as the *Rynchops* model at the same depth. To describe the process in mathematical terms, the researchers developed a fluid dynamic model incorporating drag forces that would cause energy loss as the pterosaur or bird worked against gravity to displace water with its jaw. They validated the model by showing that the drag measurements produced by the *Rynchops* and pterosaur model bills matched the estimates derived from the principles of fluid dynamics. The researchers could then use this model to estimate drag costs and potential skimming capability of other reputed skimming pterosaurs without having to make casts of their bills.

**Figure:**
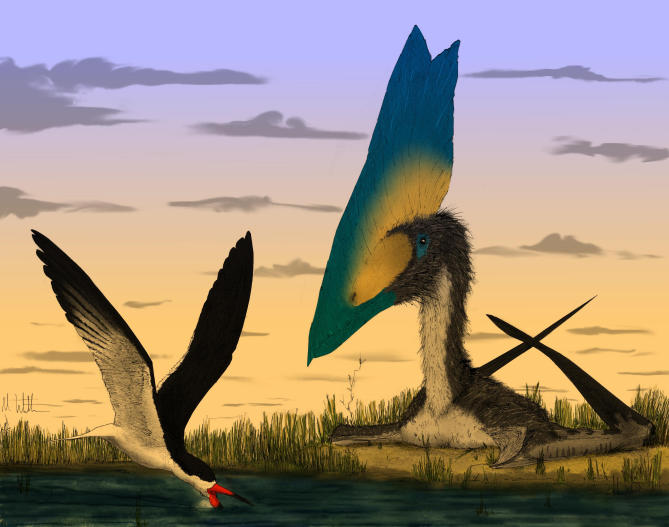
Physical and theoretical models show that pterosaurs (*Thalassodromeus*, right) could not meet the energy requirements of skimming, a rare feeding strategy practiced habitually by just a few extant *Rynchops* species (black skimmer, left). (Image: Mark Witton)

To calculate the energetic costs of skimming, the researchers compared the estimated costs of powered flight, including their estimated costs of hydrodynamic drag, to estimates of available metabolic power for self-propelled flight. (Metabolic power estimates are based on estimates of body mass and wingspan.) Skimming carried “considerably higher” flight costs for pterosaurs than for modern skimmers. But skimming is also an energetically costly foraging strategy for *Rynchops*, the researchers discovered, because their hydrodynamic drag costs are three times higher than previously estimated. Even so, the birds possess the metabolic muscle to sustain skimming. It’s unlikely, however, that pterosaurs could have mustered the power required to offset the energetic costs of skimming. (Interestingly, the researchers’ theoretical models suggest that *Tupuxuara* sp., the smaller pterosaur considered a nonskimmer based on its anatomy, could theoretically meet the energy demands of skimming, though it would have to spend half of its flight energy budget on drag.)

These results show that the bill is subjected to substantially greater hydrodynamic drag than previously appreciated, challenging the common assumption that drag costs pale in comparison to aerodynamic costs of flight. These high costs explain why skimming is habitually practiced by only three existing bird species, all *Rynchops*—and why it’s very unlikely that ancient pterosaurs fed by skimming. What’s more, the researchers argue, modern-day skimmers possess some 30 adaptations in the neck and skull to withstand hydrodynamic forces while catching prey with an immersed jaw during flight. Postulated pterosaur skimmers evince few of these adaptations, suggesting that even smaller pterosaurs with the capacity to meet skimming energy costs were not skim-feeders. While acknowledging that morphological comparisons offer clues to the ecological traits of extinct taxa, the researchers hope that others will supplement such efforts with biomechanical analyses to paint a more realistic portrait of the prehistoric landscape.

For more information on pterosaurs, go to UC Berkeley’s Museum of Paleontology Web site: http://www.ucmp.berkeley.edu/diapsids/pterosauria.html.

